# Development and Pilot Testing of the Eating4two Mobile Phone App to Monitor Gestational Weight Gain

**DOI:** 10.2196/mhealth.4071

**Published:** 2015-06-05

**Authors:** Catherine Knight-Agarwal, Deborah Lee Davis, Lauren Williams, Rachel Davey, Robert Cox, Adam Clarke

**Affiliations:** ^1^ The University of Canberra Canberra Australia; ^2^ University of Canberra Canberra Australia; ^3^ Griffith University Gold Coast Australia

**Keywords:** pregnancy, mobile phone, antenatal care, maternal obesity, intervention

## Abstract

**Background:**

The number of pregnant women with a body mass index (BMI) of 30kg/m^2^ or more is increasing, which has important implications for antenatal care. Various resource-intensive interventions have attempted to assist women in managing their weight gain during pregnancy with limited success. A mobile phone app has been proposed as a convenient and cost-effective alternative to face-to-face interventions.

**Objective:**

This paper describes the process of developing and pilot testing the Eating4Two app, which aims to provide women with a simple gestational weight gain (GWG) calculator, general dietary information, and the motivation to achieve a healthy weight gain during pregnancy.

**Methods:**

The project involved the development of app components, including a graphing function that allows the user to record their weight throughout the pregnancy and to receive real-time feedback on weight gain progress and general information on antenatal nutrition. Stakeholder consultation was used to inform development. The app was pilot tested with 10 pregnant women using a mixed method approach via an online survey, 2 focus groups, and 1 individual interview.

**Results:**

The Eating4Two app took 7 months to develop and evaluate. It involved several disciplines--including nutrition and dietetics, midwifery, public health, and information technology--at the University of Canberra. Participants found the Eating4Two app to be a motivational tool but would have liked scales or other markers on the graph that demonstrated exact weight gain. They also liked the nutrition information; however, many felt it should be formatted in a more user friendly way.

**Conclusions:**

The Eating4Two app was viewed by participants in our study as an innovative support system to help motivate healthy behaviors during pregnancy and as a credible resource for accessing nutrition-focused information. The feedback provided by participants will assist with refining the current prototype for use in a clinical intervention trial.

## Introduction

The proportion of overweight and obese pregnant women is increasing, which has important implications for antenatal care [1-3]. Of particular concern is that, compared to women who have given birth previously, young first-time mothers have a greater likelihood of gaining excess weight during pregnancy and many are entering pregnancy with a high BMI [4,5]. One recent Australian study reported that 43% of pregnant women were overweight or obese [6]. Maternal obesity and excessive GWG have well-documented associations with adverse outcomes for the woman, such as increased risk of gestational diabetes, and for the baby, such as higher risks of hypoglycemia and macrosomia [3]. High GWG has been independently associated with an increased risk of childhood obesity, suggesting that influences occurring in the fetal environment are contributing to obesity onset [7].

Various resource-intensive interventions have attempted to assist women to manage their weight gain during pregnancy with varied success. One systematic review and meta-analysis of randomized and nonrandomized controlled trials (RCTs) suggested that dietary interventions during pregnancy may be effective in decreasing total GWG. A trend toward reduced prevalence of gestational diabetes in overweight and obese women was also reported [8]. Another systematic review of RCTs concluded that the effect of providing antenatal dietary interventions for overweight or obese pregnant women on maternal and infant health outcomes remains unclear [9]. Qualitative studies show health professionals struggle to support women to manage their weight gain in pregnancy due to a paucity of resources, concern over raising the sensitive issue of weight, the fact that weighing women in pregnancy is not routine, and a lack of high level guidance [10].

More recently, a systematic review examined new communication technologies and their potential to support lifestyle interventions during pregnancy [11]. The authors concluded that there is a paucity of data and RCTs examining the effectiveness of communication technology with pregnant women, particularly among those who are overweight and obese. A mobile phone app has been proposed as one such convenient and cost-effective approach to maternal obesity interventions. Mobile phones are ubiquitous in Australia, with an estimated 8.67 million users. Mobile app downloads increased by 85% over a 12-month period from 2.42 million users downloading a mobile app in June 2011 to 4.45 million in June 2012 [12].

A wide range of apps are available that focus on general weight management. Hebden et al developed and qualitatively evaluated a series of apps aimed at modifying lifestyle behaviors associated with excess weight gain in young adulthood [13]. Lee et al developed a mobile phone-based diet game for weight control [14] and Bexelius et al formulated an app for monitoring physical activity [15]. Hearn et al developed a mobile phone app and accompanying website for perinatal women to help track their weight, diet, physical activity, emotional well-being and sleep patterns [16]. After careful review of both Android and iPhone apps, the researchers identified very few that focused specifically on weight management in pregnancy and none, to our knowledge, that contained a visual graphing function for women to input and follow their GWG. The aims of this study were to: (1) develop an app providing real-time feedback on gestational weight gain progress and credible, up-to-date nutrition advice for pregnancy; and (2) identify any usability problems with the app and determine participant satisfaction with the product.

## Methods

### Development of the Eating4Two App

**Figure 1:**
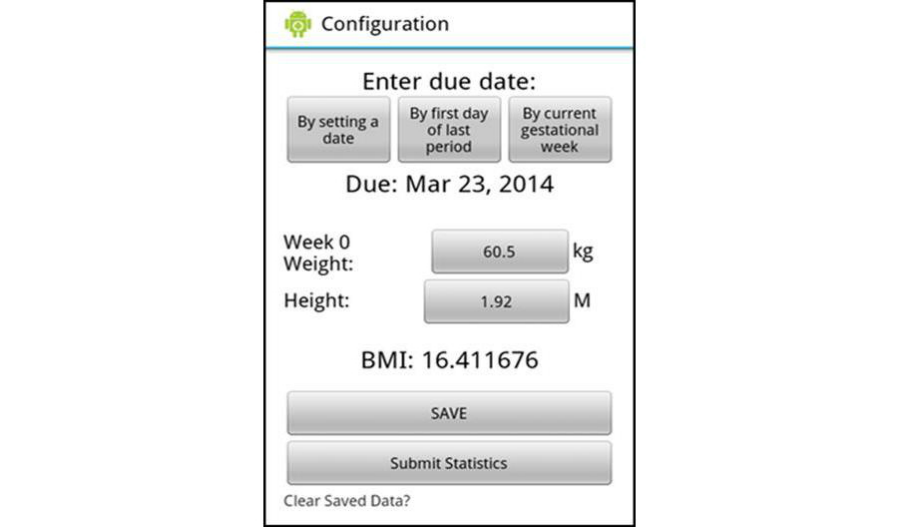
Configuration screen.

**Figure 2:**
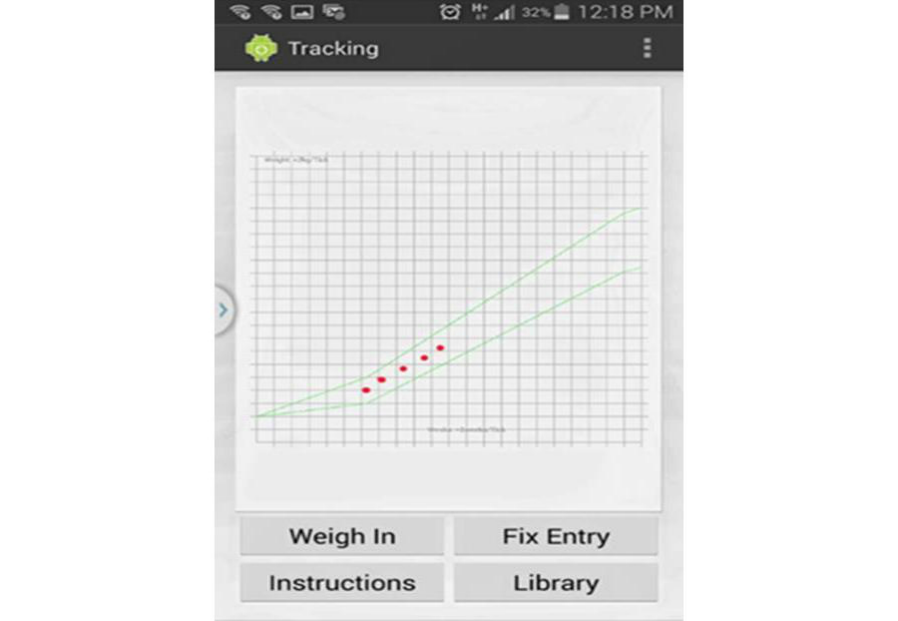
Gestational weight gain calculator screen.

The overall content and usability for the Eating4Two app was shaped by qualitative investigation and supported by an evidence-based approach. The study team first met in August 2013 to discuss the project plan and an IT specialist was employed shortly after to write the software program for the app. It was decided that an Android 2.1 and higher platform be used as this was the most cost-effective choice [17]. The study team met on a weekly basis in order to resolve issues and review the progress of the project. A simple, visual graphing function that allows the user to configure their start BMI, estimate due date, and weigh in throughout the pregnancy and that provided real-time feedback was developed as part of the app. The opening screen of the app requires users to agree to the statement that the app does not replace the care and advice provided by a health professional. In order to progress to the app functions, the user must indicate that they agree with the statement. Alternatively, they are given the option to exit. Once the “I agree” tab has been pressed, users enter the configuration screen shown in Figure 1. Gestation (in weeks) is calculated by setting a date (usually provided by ultrasound), from first day of last menstrual period, or by current gestational week. The next step requires input of weight and height in either metric or imperial units. To appeal to a broad range of potential consumers, BMI is automatically calculated and appears on the screen (BMI is the division of weight in kilograms by height in meters squared). Once the “Save” tab has been pressed, the recommended GWG is calculated to align with Institute of Medicine guidelines based on BMI at conception. This function allows the user to check, at any time during their pregnancy, where they are positioned on the graph in terms of weight gain (see Figure 2). Numbers on the graph were purposely omitted to reduce weight-related stress as appropriate trends within each participant’s recommended weight gain range were felt to be more important than numbers aiming for an exact weight.

**Figure 3:**
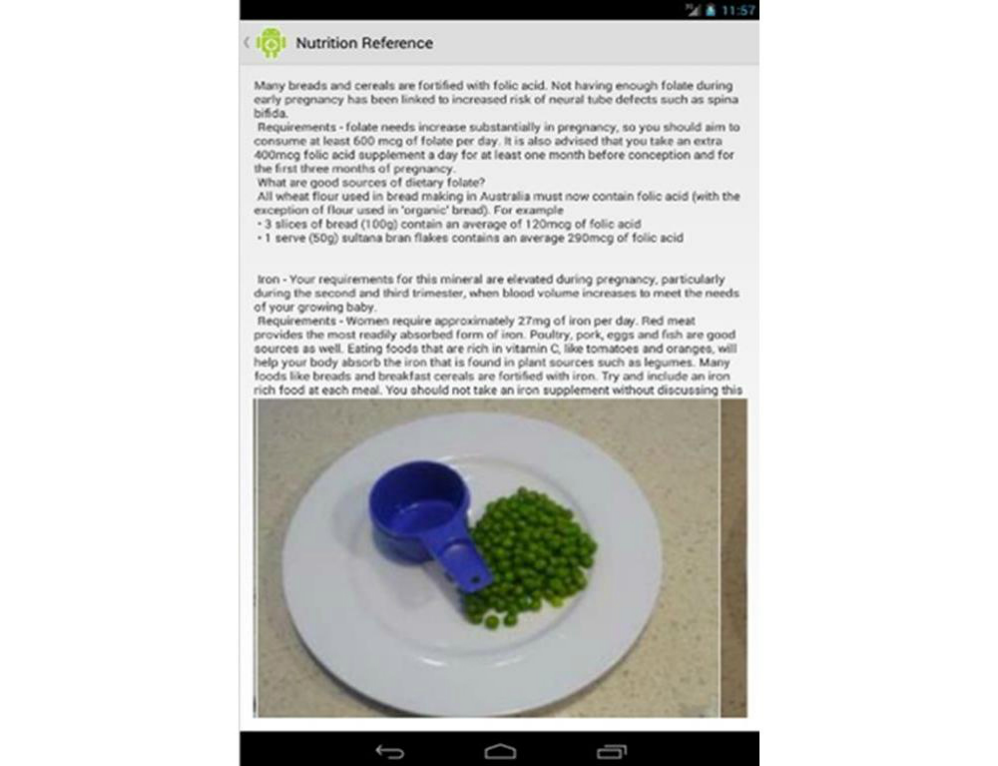
Example of nutrition library screen.

Another function, the “Library” tab, contains information about nutrients, foods, menus, behaviors, and symptoms especially relevant to pregnancy (see Figure 2). This information was drawn from the Commonwealth Government of Australia’s National Health and Medical Research Council [18,19], the Dietitian’s Association of Australia [20], and Food Standards Australia and New Zealand [21]. Photographs demonstrating recommended food portions provide a visual guide of what a “standard serving” looks like; for example, a half a cup of green peas [22] (see Figure 3). All 5 food groups are represented [18,19].

**Table 1:** Dietary information contained in the Eating4Two app library.

Topic	Information under this topic
General dietary intake	Healthy eating advice (eg, dieting while pregnant discouraged). However, there is no need to eat double than usual.
Alcohol	The potential risks of consuming alcohol during pregnancy are outlined. There is no known completely safe level of alcohol consumption during pregnancy.
Energy	During the first trimester, energy (kJ) intake should remain about the same as it was prior to pregnancy. Second and third trimester energy (kJ) should increase by about 600 kJ/day. Practical examples of how to achieve this were provided.
Iron	During pregnancy, requirements for this mineral are elevated. It is recommended that women consume approximately 27 mg/day. Practical dietary examples of how to achieve this were provided.
Folate	During pregnancy, requirements for this vitamin are elevated. It is recommended that women consume approximately 600 mcg/day (plus a 400 mcg supplement/day). Practical dietary examples of how to achieve this were provided.
Calcium	During pregnancy, requirements for this mineral are elevated. It is recommended that women consume approximately 1,000 mg/day. Practical dietary examples of how to achieve this were provided.
Iodine	During pregnancy, requirements for this mineral are elevated. It is recommended that women consume approximately 200 mcg/day. Practical dietary examples of how to achieve this were provided.
Zinc	This mineral is widely available from a variety of foods, making it possible for pregnant women to achieve their requirements through a balanced diet alone.
Nausea	Commonly experienced in the first trimester. Always discuss symptoms with health care provider. Nutrition-related information to relieve nausea (eg, eat small amounts of food often) was provided.
Heartburn	Commonly experienced in the third trimester. Always discuss symptoms with health care provider. Nutrition-related information to relieve heartburn (eg, stay upright after eating) was provided.
Constipation	May occur at any stage during pregnancy. Always discuss symptoms with health care provider. Nutrition-related information to relieve constipation (eg, drink plenty of fluids) was provided.
Tiredness	May occur at any stage during pregnancy and for some women may be ongoing. Nutrition-related information to assist women (eg, suggestion to make a batch of meals all at once and have a ready supply in the freezer) was provided.
Listeria	A bacterium that can contaminate food and cause listeriosis. It’s a rare infection, but it’s very serious if contracted during pregnancy. Dietary information was included on what to avoid.
Mercury	Fish is an important part of a healthy diet. Some types of fish may contain mercury. Practical examples of what and how much fish is ideal to eat during pregnancy were provided.
Cravings	May be driven by hormonal changes. Unlikely to be the body’s way of indicating a nutrient deficiency exists. Discuss unusual or inappropriate cravings with health care provider. Healthy tips on dealing with cravings (eg, cravings for ice cream may be satisfied by eating frozen berries) were provided.
Serving sizes	Photographs of recommended serving sizes (eg, nuts, lean meat, bread) were included.

### Stakeholder Review

A stakeholder group was established at the end of August 2013 to further inform development. This group included the researchers, an obstetrician, and 4 women of child-bearing age. In mid-September 2013, the stakeholder group met and reviewed existing dietary and antenatal care apps plus a preliminary draft of the Eating4Two app. Stakeholders provided invaluable feedback on the app’s content and functionality. The first version of the app was finalized in November 2013.

### Pilot Testing the Eating4Two App

Ethical approval for the evaluation of the app was sought and obtained from the University of Canberra Human Research Ethics Committee (ref: 13–173). An Eating4Two study information flyer was circulated via the university intranet and on a local mother’s group Facebook page. Women were eligible to participate if they were at no more than 30 weeks gestation, had a singleton uncomplicated pregnancy, were 18 years or older, and were able to attend meetings at the university. Ten pregnant women were recruited and invited to attend an Eating4Two introductory session at the University of Canberra. The first part of the session included both verbal and written information explaining the background and objectives of the study. Women were then invited to provide signed consent. The second part of the session involved downloading the app to each woman’s mobile phone and providing a practical demonstration on how to use it. All women owned and regularly used mobile phones. However, 50% of the study participants had access to an iPhone. The University of Canberra supplied these women with a prepaid Huawei Ascend Y300 (powered by Android) for the duration of the 6-week evaluation period. Participants were asked to weigh themselves at the same time every day and on the same day each week (ie, Friday morning at 7) using the same bathroom scale for each weigh-in and to insert the result immediately into the app. No more than 1 weigh-in per week was recommended since researchers wanted to prevent angst and weight-related stress. Once weight was measured, women were encouraged to insert this immediately into the app. They were encouraged to view the nutrition information as desired. Participants were provided with contact technical support in the event they required assistance. Women began using the app from late November 2013 to early January 2014. We provided each participant with a AUD $100 gift voucher to compensate them for their time. Women were not informed of the gift vouchers until after they had agreed to participate in the study.

### Evaluation process

An online survey that focused on usability was emailed to all participants midway through the evaluation period. The first 2 questions were:

1. Have you found it useful to graph and monitor your weight change with the app?
2. What do you think about the nutrition information contained in the app?

Participants were asked to provide responses on a 5-point Likert scale [23] where 1 means very unhelpful and 5 means very helpful.

Questions 3, 4, and, 5 were open-ended and asked:

1. What do you like best about the app?
2. What do you like least about the app?
3. Have you any additional comments regarding the mobile phone app?

At the end of the evaluation period, 2 focus groups (FG) [24] and 1 individual interview were conducted. FG1 (n=6), FG2 (n=3), and individual interview (n=1) were held in January, February, and March 2014, respectively. A facilitatory style was employed, which included the use of verbal and nonverbal cues and sought not to influence answers. Each focus group and the individual interview gave participants the opportunity to add any information relating to the topic that may have been missed. Sessions were audio recorded and supported by written notes. The focus group discussions and the individual interview were transcribed verbatim and entered into a word processing document.

The open-ended comments from the survey and transcripts were analyzed using inductive thematic analysis, which allows findings to emerge from those that dominate in the raw data. The primary aim of this type of analysis is to establish clear links between the research objectives and the research findings and to ensure these links are both transparent and justifiable [25,26].

## Results

### Participants

Ten women living in or close to Canberra, Australia, participated in the pilot. Participants were 18 years of age or older with a singleton, uncomplicated pregnancy. All participants provided signed consent and were given unique numbers to ensure anonymity (see Table 2).

**Table 2:** Demographic information of participants at the beginning of the 6-week pilot study.

Participant identification No.	Gestation, No. of weeks	Parity	Prepregnancy BMI
1	17	0	Not provided
2	19	0	Not provided
3	19	1	20.5
4	30	0	23.8
5	13	0	24.5
6	15	0	Not provided
7	17	0	23.9
8	22	1	23.3
9	30	1	22.1
10	19	1	26.1

### Survey

For the first question, 70% (n=7) of participants reported finding the app a helpful tool to graph and monitor their weight change, 20% (n=2) found it neither helpful nor unhelpful, and 10% (n=1) found it unhelpful (mean=3.6; median=4). For the second question, 10% (n=1) of participants reported finding the nutrition information contained in the app very helpful, 50% (n=5) found it helpful, 30% (n=3) neither helpful nor unhelpful, and 10% (n=1) found it unhelpful (mean=3.6; median=4). Women acknowledged that the Eating4Two app is a motivational tool that is generally simple to use. Women reported that they would have liked scales or other markers on the graph to demonstrate exact weight gain and that the nutrition information should be formatted in a more user friendly way.

### Focus Groups and Individual Interview Findings

#### Overview

Participants provided very similar feedback to the online survey. Women found the Eating4Two app to be a motivational tool but would have liked more explicit feedback regarding their GWG. They liked the nutrition information, which included food photos demonstrating portion sizes. However, participants felt that the food photos should be clearer with labels attached and that the nutrition library should be made easier to navigate. Overall, 3 themes relating to user experience and acceptability were identified and are presented below.

#### Theme 1: Functionality Is Important When Navigating an App.

Most participants described the Eating4Two app as “very user friendly” [FG1, P6] and viewed its function as being an efficient and convenient way to monitor GWG. As 1 woman stated:

It takes 3 seconds to input the data every couple of days. . . . I suppose I am looking at the effort verses reward, which is actually quite good

FG1, P1

Whereas other women expected the app to require less user input, saying:

It takes 2 presses on the screen to bring the numbers up when you type your weight in . . . once to click in the box and another to bring up the numbers. It would have helped to have the numbers come up automatically.

FG2, P9

Women expressed a desire to know exactly how their weight was tracking. One participant commented:

I would have at least liked scales on the graph—that would have been good.

FG2, P7

This was supported by another woman, who said:

A little feedback from what I was putting in would have been helpful . . . so you know what the number range is in the projection that it gives you because you have no concept of whether you are 1 kilo below the line or 2 or 5 or 10.

FG1, P3

The written and visual information regarding quantity and type of foods to eat during pregnancy was seen to be an important inclusion. One woman commented that “the serving sizes . . . having that information really helped” [FG1, P6] and another woman described this function as a unique feature, saying:

I haven’t seen another app or even just literature that describes usually what a standard serving size is supposed to look like . . . to see it, I thought was really good.

FG2, P8

However, other participants questioned the clarity of photos, with 1 saying:

I didn’t actually find them that easy to gauge the serving sizes or sometimes what food I was looking at.

FG1, P3

Participants suggested that the nutrition component could be improved by inserting a label under each photo stating quantity and type of food and by presenting the information “in categories and on different pages” [FG1, P1] instead of in 1 scroll-down sheet. Participants suggested additional functions that may improve the usability of the Eating4Two app; for example, a routine pop-up message “as a reminder of the day to weigh in” [FG2, P7] or a prompt to bring up conversations with a health care provider about any weight- or nutrition-related issues.

#### Theme 2: the Eating4two App Helps Motivate Women to Be Healthy During Pregnancy.

Women expressed motivation to stay healthy during pregnancy. TheEating4Two app was viewed as a type of support system to help promote motivating behaviors, as 1 participant stated:

I think it was really good as it encouraged me to keep on track.

FG1, P3

The plethora of dietary information available was seen as confusing, but the app simplified this process. For 1 woman this was especially true, she said:

I think I most struggle with things I can’t eat. People present you with food like ahhh, can I eat that? So I found that [app function] quite handy

FG1, P5

This woman emphasized that the app not only motivated her to make healthy food choices but also provided her with reassurance that the choices she did make were appropriate.

Despite maternal obesity being a growing problem, health care practice has moved way from weighing pregnant women in recent years [10].The participants confirmed this, stating that they were not weighed as part of routine antenatal care. Using the app motivated them to self-monitor their weight progress and, for some, the app encouraged them to initiate conversations with their health care providers regarding their gestational weight gain, with 1 woman saying:

When I noticed there wasn’t any gain for ages . . . I had occasion to raise it

FG2, P8

One woman commented on the variability of weight over a day and its influence on when she weighs in, saying:

The time of day I weigh myself really affects whether my weight is in the recommended range. So I find myself cheating, like weighing myself first thing in the morning more often to feel better about my weight so it is in the normal range. . . . My weight will easily fluctuate 2-3kg a day based on fluid retention

Individual interview, P10

This participant offered the suggestion that weight gain results may be more helpful if they were color-coded according to morning, noon, or night measures.

#### Theme 3: How Far Can an App Go?

Pregnancy is a time when many women develop a strong desire to learn as much as they can about keeping healthy for the sake of themselves and their unborn baby [27]. Nevertheless, there is a huge amount of pregnancy-related information available, particularly via the World Wide Web, which can sometimes be confusing and contradictory. As 1 participant commented:

I have put myself on a self-imposed, strictly no googling policy. . . . I have a sore finger, I have a brain tumor . . . that is the sort of thing Google will tell you. All my information about diet and things has come from a 1-page printout that my GP gave me . . . and from what’s in the [Eating4Two] app.

FG1, P1

This comment raises the issue of credibility. Some women felt more comfortable obtaining information from sources deemed reputable—such as from their doctor, midwife, or university—rather than from “other apps” or websites where little or no information is provided regarding authorship or institution of origin, etc.

Other participants were happy to access a wide variety of different resources. One woman admitted to trying out a number of pregnancy apps in addition to the Eating4Two app. Apart from ours, the apps she continued to use had certain features that she found engaging, such as:

One app doubles the quiz for you each week . . . questions are dependent on what week [of pregnancy] you are at so it will be like, oh, this week is really important that you do this. . . . It will say, “Hey you can improve your score this week if you do this much extra exercise.”

FG1, P4

Some women felt that the information contained in the Eating4Two app was not comprehensive enough and needed to include more direct reference to the peer review literature and commentaries from experts in the field, saying:

Personally, I need a lot more information in there to be able to recommend it to someone else. . . . [I]f there is sort of references . . . to professionals in the area, a lot more detail then yes, you know, I would recommend it.

FG1, P2

Nevertheless, most women expressed a desire to continue using the Eating4Two app post-evaluation. Participants enjoyed using the Eating4Two app and felt it was a valuable adjunct to routine antenatal care, but that it required some refinements in order to improve usability and make the information it contains more informative. One woman stated:

The information provided could have related a bit more to where you were on the [weight] scale. So, if you were a bit under, then there could be a section on evaluat[ing] your eating or something that could link the information with the graph.

FG2, P7

Participants acknowledged that the Eating4Two app should be used in conjunction with traditional modes of antenatal care. However, there were differing views regarding the type and quantity of information that a maternity app should contain and what functions it should or shouldn’t claim to deliver. One participant felt that such apps had the potential not only to provide clinical input but should be personally reassuring, she commented:

When someone is pregnant they want reassurance . . . the fact that someone may be a bit or way over the line for me means nothing, but it would mean a lot more if there is backup.

FG1, P2

Others recognized that every woman is different and that each pregnancy has its own unique challenges. However, they were very clear in their beliefs that front-line advice should be obtained from a doctor or midwife. One woman stated:

I think you still have to appreciate that it is an app and it can’t possibly replace health care advice [from a health professional].

FG1, P1

## Discussion

### Overview

Evaluation of the usability of mobile phone apps is crucial for success, so developers can adapt and improve them in the rapidly changing world of mobile technology. However, very few studies have been published which focus on the development and use of mobile phone apps for individual dietary change or weight gain monitoring. None to our knowledge have reported on pregnancy apps though a number of studies testing the efficacy of mobile phone apps are currently in progress [27,28]. Hebden et al developed and tested 4 mobile phone apps aimed at improving nutrition and physical activity lifestyle behaviors during young adulthood. Qualitative feedback provided little suggestion for content change in these apps, with the major concerns being slow running speed and requirement to log in to the apps [13]. In another qualitative investigation, Dennison et al reported that healthy young adults displayed some interest in using health care apps that support behavior change. Legitimacy, effort required, and immediate effects on mood emerged as important influences relating to app usage [29].

Our study reinforced these results plus found that women were interested in not only learning new information but in recording, and having as a reference, the exact details of their gestational weight status. Wennberg et al described pregnant women as having an eagerness to learn everything about pregnancy and seeking that information from multiple sources including health care providers, friends, magazines, and the Internet [30]. Some participants in the Eating4Two pilot test accessed information from only a few sources, whereas others admitted to accessing many. Interestingly, women in the current study expressed a desire for the Eating4Two app to contain more prompts as a reminder to undertake certain activities such as weighing in and pop-up messages that provide support but include action-oriented dialogue when it is required. In contrast, Dennison et al and Hebden et al both reported in that users of mobile phone apps often expressed irritation at receiving alerts and some became demotivated by viewing records that showed they were not meeting a goal [29,30].

Tripp et al emphasized that mobile phone apps have the potential to influence health behaviors in expectant women by providing almost-anywhere, at-any-time interactive and often personalized information at the push of a button. Due to the increasing popularity of apps, particularly among women of child-bearing age, they acknowledge that traditional antenatal services need to accommodate the use of such technology [31]. The participants in our study reported that they were not weighed as part of routine antenatal care, so the Eating4Two app provided them with the opportunity to self-monitor GWG. The fact that this led some women to initiate conversations with their health care providers regarding appropriate GWG and pregnancy-related nutrition, suggests that the app has the potential to improve nutrition related quality of care.

Dennison et al reported that users of behavior-change apps may lose motivation over time and engage in only transient use. Whether this view is applicable to pregnant users remains to be seen [29]. There is evidence that pregnant women are motivated to keep healthy not only for themselves but their unborn baby. Verbeke and De Bourdeaudhuj found that pregnant women had higher intakes of fruit and dairy products and reduced consumption of foodstuffs deemed to have elevated safety risks than nonpregnant women [32]. A study by Szwajcer et al concluded that pregnancy could indeed be a life-changing event leading to increased nutrition awareness and could influence future dietary-related behaviors [33]. Conversely, Shub et al found that women in their study had poor knowledge of GWG recommendations and the complications associated with excess weight gains [34]. The sensitive nature, and sometimes avoidance, of weight-related discussions between a client and their maternity caregiver has been suggested as 1 reason for this lack of knowledge [10]. As a result, opportunities for healthy lifestyle education, at what is an opportunistic time for many women, are missed.

Bridging gaps in knowledge is an important step toward improving outcomes for both women and offspring. Encouraging the use of apps like Eating4Two is 1 way this may be achieved. In saying this, Tripp et al has acknowledged that the proliferation of health apps may reduce pregnant women’s reliance on health care professionals for advice [31]. There are obviously potential risks with this scenario. It is at this point that we should provide a rationale for our choice of data collection methods. Both focus groups and individual interviews are techniques relying on qualitative methodology. Focus group discussions allow multiple perspectives to be heard and participants may feel encouraged to share their experiences in a homogenous group [35]. While we would have preferred only to use focus groups, 1 participant was unable to attend either focus group discussion. Rather than miss out on her feedback, we used the identical protocol to interview her individually. During the analysis stage, we were careful to consider any potential differences between responses provided by members of the focus group discussions and the individual participant interviewed. No new themes arose from the data collected from the individual participant, thus 1 method corroborated the other. Even though it was not initially planned this way, the use of both methods (and resulting corroboration) does serve as a form of triangulation [24,25,35].

### Limitations

There are some limitations that must be acknowledged. This study contained only a small sample size of women, thus limiting the validity of results. Their views may not reflect those of pregnant women elsewhere. The pre-pregnancy BMIs we obtained were self-reported and are only an estimate of weight status. We did not collect participant’s age or educational status, which is a potential limitation of the study. However, the aim of our research was to test usability of the Eating4Two app in women of childbearing age. Therefore, regular use of a mobile phone and being pregnant were deemed to be the most useful demographics to collect. It is also important to point out that the Eating4Two app has been designed as an adjunct to usual maternity care and in no way should replace the individual advice provided by a health professional.

### Conclusion

TheEating4Two app was viewed by the majority of participants in our study as a support system to help motivate healthy behaviors during pregnancy and as a credible resource for accessing information, with the GWG graphing function perceived as a unique and useful feature. However, women wanted the Eating4Two app to display absolute figures rather than ranges, to include more comprehensive pregnancy-related resources, and be formatted in a more user friendly fashion. This feedback will assist with the refinement of the current prototype and will be used as part of a pilot RCT of pregnant women.

## Abbreviations

BMI: body mass index
FG: focus group
GWG: gestational weight gain
P: participant
RCT: randomized controlled trial
